# HIV-infected persons with type 2 diabetes show evidence of endothelial dysfunction and increased inflammation

**DOI:** 10.1186/s12879-017-2334-8

**Published:** 2017-03-29

**Authors:** Malene Hove-Skovsgaard, Julie Christine Gaardbo, Lilian Kolte, Kamilla Winding, Ingebjørg Seljeflot, Asbjørn Svardal, Rolf Kristian Berge, Jan Gerstoft, Henrik Ullum, Marius Trøseid, Susanne Dam Nielsen

**Affiliations:** 1 Department of Infectious Diseases, Rigshospitalet, University Hospital of Copenhagen, opgang 86, 3 sal, Blegdamsvej 9, 2100 Kbh Ø, Copenhagen, Denmark; 2 Department of Infectious Diseases, Hvidovre Hospital, University Hospital of Copenhagen, Copenhagen, Denmark; 3 Centre of Inflammation and Metabolism, Rigshospitalet, University Hospital of Copenhagen, Copenhagen, Denmark; 4 Center for Clinical Heart Research, Department of Cardiology, Oslo University Hospital Ulleval, Oslo, Norway; 5 Faculty of Medicine, University of Oslo, Oslo, Norway; 6 Department of Clinical Science, University of Bergen, N-5020 Bergen, Norway; 7 Department of Heart Disease, Haukeland University Hospital, N-5021 Bergen, Norway; 8 Department of Clinical Immunology, Rigshospitalet, University Hospital of Copenhagen, Copenhagen, Denmark; 9 Section of Clinical Immunology and Infectious Diseases, Oslo University Hospital Rikshospitalet, Copenhagen, Norway; 10 Research Institute of Internal Medicine, Oslo University Hospital, Oslo, Norway; 11 Department of Infectious Diseases, Oslo University Hospital, Oslo, Norway; 12 K.G. Jebsen Inflammatory Research Center, University of Oslo, Oslo, Norway

**Keywords:** HIV infection, Type 2 diabetes, Inflammation, ADMA, TMAO

## Abstract

**Background:**

Increased incidence of cardiovascular diseases (CVD) in both HIV infection and type 2 diabetes (T2D) compared to the general population has been described. Little is known about the combined effect of HIV infection and T2D on inflammation and endothelial function, both of which may contribute to elevated risk of CVD.

**Methods:**

Cross-sectional study including 50 HIV-infected persons on combination anti-retroviral therapy (cART), with HIV RNA <200 copies/mL (*n* = 25 with T2D (HIV + T2D+), *n* = 25 without T2D (HIV + T2D-)) and 50 uninfected persons (*n* = 22 with T2D (HIV-T2D+) and *n* = 28 without T2D (HIV-T2D-)). Groups were matched on age and sex.

High sensitive C-reactive protein (hsCRP) was used to determine inflammation (cut-off 3 mg/L). The marker of endothelial dysfunction asymmetric dimethylarginine (ADMA) was measured using high performance liquid chromatography. Trimethylamine-N-oxide (TMAO), a microbiota-dependent, pro-atherogenic marker was measured using stable isotope dilution LC/MS/MS.

**Results:**

The percentage of HIV + T2D+, HIV + T2D-, HIV-T2D+, and HIV-T2D- with hsCRP above cut-off was 50%, 19%, 47%, and 11%, respectively. HIV + T2D+ had elevated ADMA (0.67 μM (0.63-0.72) compared to HIV + T2D- (0.60 μM (0.57-0.64) *p* = 0.017), HIV-T2D+ (0.57 μM (0.51-63) *p* = 0.008), and HIV-T2D- (0.55 μM (0.52-0.58) *p* < 0.001). No differences in TMAO between groups were found. However, a positive correlation between ADMA and TMAO was found in the total population (r_s_ = 0.32, *p* = 0.001), which was mainly driven by a close correlation in HIV + T2D+ (r_s_ = 0.63, *p* = 0.001).

**Conclusion:**

Elevated inflammation and evidence of endothelial dysfunction was found in HIV-infected persons with T2D. The effect on inflammation was mainly driven by T2D, while both HIV infection and T2D may contribute to endothelial dysfunction. Whether gut microbiota is a contributing factor to this remains to be determined.

**Electronic supplementary material:**

The online version of this article (doi:10.1186/s12879-017-2334-8) contains supplementary material, which is available to authorized users.

## Background

Introduction of combination anti-retroviral therapy (cART) has increased life expectancy for HIV-infected persons. However, new challenges are emerging, and incidence of cardiovascular disease (CVD) is increased in HIV-infected persons compared to the general population [1]. Chronic inflammation resulting in endothelial dysfunction and atherosclerosis may contribute to increased risk of CVD in HIV infection [2].

Type 2 diabetes (T2D) is also characterized by chronic inflammation and endothelial dysfunction [3], and T2D is an independent risk factor for CVD in the general population as well as in the HIV-infected population [3, 4]. In HIV infection, it is debated if prevalence of T2D is higher than in the general population [5–7]. Traditional T2D risk factors including age and obesity as well as HIV-related factors such as some cART regimes and chronic inflammation are found to be associated with development of T2D in HIV-infected populations [8–10]. Thus, with an aging HIV-infected population and an emerging worldwide diabetes epidemic, a combination of HIV infection and T2D may become a clinical challenge.

Asymmetric dimethylarginine (ADMA) is elevated in both HIV infection and T2D [11, 12]. ADMA is an endogenous inhibitor of endothelial NO synthase and a well-established marker of endothelial dysfunction [13]. L-Arginine is the natural precursor for NO in the endothelium. L-arginine/ADMA ratio is important since ADMA, by a competitive reversible blocking of NO-production from L-arginine, affects endothelial function [13, 14].

The gut microbiota is altered in both HIV infection and in T2D [15, 16], and studies suggest that altered microbiota is associated with increased inflammation [17, 18]. Recently, trimethylamine-N-oxide (TMAO) a metabolite from dietary carnitine and choline was found to promote atherosclerosis and predict CVD in persons with T2D as well as HIV-uninfected cohorts [19, 20]. Production of TMAO seems to be dependent on metabolism by gut microbiota, as one week of antibiotics is sufficient to block its formation [19, 21, 22]. Since the gut microbiota is altered in both HIV infection and in T2D [15, 16] elevated TMAO may contribute to inflammation and endothelial dysfunction in these patients.

Little is known about the combined effect of HIV infection and T2D on inflammation and endothelial function. The aim of this study was to determine if concurrent T2D in HIV-infected persons results in higher levels of inflammation and endothelial dysfunction compared to either HIV infection by itself or T2D in uninfected persons. We hypothesized that higher inflammation and elevated markers of endothelial dysfunction would be found in persons with both HIV and T2D compared to persons with either HIV or T2D. TMAO was included in our study to explore a possible link between gut microbiota, inflammation and endothelial function in patients with HIV infection and T2D. We determined inflammation, endothelial dysfunction, and the potential contribution of gut microbiota by measuring high sensitive C-reactive protein (hsCRP), ADMA, L-arginine, and TMAO in HIV-infected persons with T2D (HIV + T2D+) and three control groups: HIV-infected persons without T2D (HIV + T2D-), uninfected persons with T2D (HIV-T2D+), and healthy controls (HIV-T2D-).

## Methods

### Participants

We performed a cross-sectional study including 100 participants (25 HIV + T2D+, 25 HIV + T2D-, 22 HIV-T2D+, and 28 HIV-T2D-). ADMA was the main outcome in this study. In a previous study ADMA was determined in HIV-positive individuals, mean (standard deviation) was 0.59 μM (0.31, 23]. In that study, individuals with T2D were not included. However, in other studies in uninfected individuals with metabolic syndrome or T2D and 48% and 130% increase in ADMA was found compared to controls [12, 24]. Assuming the same difference between HIV-infected individuals with and without T2D (0.59 μM vs. 0.87 μM), power = 0.8, and alpha = 0.05, a minimum of 20 individuals are needed in each group. We included at least 22 individuals in each group. Inclusion criteria for HIV-infected persons were treatment with cART and suppressed viral replication (HIV RNA < 200 copies/mL.) Inclusion criteria for persons with T2D were confirmed T2D with one or more of the following: HbA1c > 48 mmol/mol (2 tests), fasting blood glucose >7 mmol/l (2 tests), or 2 h blood glucose level on >11.1 mmol/L after a glucose tolerance test. Also, all persons with T2D were treated with diet and/or oral anti-diabetics and/or insulin. All persons without T2D should have normal fasting glucose (<6.1 mmol/L) and HbA1c < 48 mmol/mol. Exclusion criteria were immunosuppressive treatment, acute infections, malignancy, and pregnancy. All HIV-infected persons with T2D attending regular controls at the Department of Infectious Diseases at University Hospital of Copenhagen, Rigshospitalet or Hvidovre Hospital, and who fulfilled inclusion and exclusion criteria, were invited to participate in the study. Participants in the three control groups were recruited in order to match for age and sex. Inclusion was stopped when at least *n* = 22 in each group and a total of *n* = 100 was reached.

Patients with T2D were included from the Department of Endocrinology, and Center of Inflammation and Metabolism, University Hospital of Copenhagen, Rigshospitalet. Healthy controls were recruited among hospital staff. All HIV-positive participants included in the study had a confirmatory positive HIV test. A negative HIV test was not performed for participants in the uninfected control groups, since the prevalence of HIV in Denmark is 0.1%, and it seems reasonable to assume that clinical healthy participants are HIV-negative. Six HIV-T2D+ also participated in another study concerning the effect of short duration, high-intensity interval training on endothelial function and metabolism (on going). Participants were included before training.

The Framingham Risk Score was calculated based on gender, age, height, weight, current smoking status, diabetes, ECG-left ventricular hypertrophy, systolic blood pressure, total cholesterol, and HDL. The calculation was performed using Risk Assessment Tool System available by Centre of Excellence for Health, Immunity and Infectious Diseases (CHIP) [25].

The study was performed in accordance with the Declaration of Helsinki and approved by the local ethical committee (H-4-2012-076 CIM VEK) and the Danish Data Protection Agency.

### Laboratory analyses

Fasting blood samples were collected from all participants. HIV RNA was measured in HIV-infected persons and glucose, HbA1c, CD4+ and CD8+ count were measured in all participants as routine analyses at the time of inclusion. HsCRP was determined using immunoturbidimetric analysis (Tina-quant hsCRP latex assay, Roche/Hitachi, Cobas, Mannheim, Germany). Data on hsCRP were only available on 87 participants. A cut-off at 3 mg/L was used to assess high risk of CVD [26].

ADMA and L-arginine were measured in snap-frozen EDTA plasma by high performance liquid chromatography and precolumn derivatization with o-phthaldialdehyde (Sigma Chemicals Co, St. Louis, MO, USA) as described [27]. Inter-assay CV’s were <5% for both assays.

Stable isotope dilution liquid chromatography with tandem mass spectrometric (LC/MS/MS) was used for quantification of TMAO. TMAO was monitored in positive multiple reaction monitoring (MRM) MS mode using characteristic precursor-product ion transitions: m/z 76 → 58 as described [22]. All stable isotope labeled internal standards were from Cambridge Isotope Laboratories, Inc., Andover, MA, USA.

### Statistical analyses

Data were tested for normal distribution, and logarithmic transformation was used as appropriate. Results are given as mean and 95% Confidence Interval (95% CI) or geometric mean (95% CI). Differences between groups were analyzed using one-way ANOVA and t test. Correlations were done using Pearson correlation. Pearson chi-square test was used on categorical data. Furthermore, potential predictors of endothelial dysfunction were investigated in a linear regression analysis. HsCRP and TMAO were not normally distributed and therefore log-transformed before analyses. Two-tailed *p*-values <0.05 were considered significant. Statistical analyses were performed using SPSS version 20 (SPSS, Inc.; Chicago, IL, USA) and GraphPad Prism 5 (GraphPad Software, San Diego, CA, USA).

## Results

### Study population

Characteristics of study participants are displayed in Table 1. There was no difference in age or sex between the groups. The HIV + T2D+ and HIV + T2D- groups had comparable CD4+, CD8+ and nadir CD4+ cell counts, HIV RNA, treatment duration, and time since HIV diagnosis. However, Body Mass Index (BMI) was higher in HIV-T2D+ compared to other groups. The Framingham Risk Score was used calculating the 10-year risk of CVD in the four groups [25]. As expected a significant difference between groups was found (Table 1). However, since diabetes is included in The Framingham Risk Score calculation, the groups with identical diabetes status were compared. The calculated 10 years risk of CVD did not differ between HIV + T2D+ and HIV-T2D+ or between HIV + T2D- and HIV-T2D- (Table 1).

**Table 1:** Characteristics of the study population. Data presented as mean and 95% CI.Differences between groups are analyzed using one-way ANOVA or Pearson chi-square test. Results are given as mean and 95% Confidence Interval. a, b, c, d are analyzed using t-test; a: *p* > 0.05 vs. HIV + T2D-, b: *p* < 0.05 vs. HIV + T2D-, c: *p* > 0.05 vs. HIV-T2D+, d: *p* < 0.05 vs. HIV-T2D+. BMI: Body Mass Index

N	HIV + T2D+	HIV + T2D-	HIV-T2D+	HIV-T2D-	P
	25	25	22	28	
Age (years)	58 (55–61)	55 (50–58)	57 (53–60)	57 (55–60)	0.638
Gender (% male)	92	96	89	72	0.079
CD4 count (cells/μL)	672 (554–790)^a^	663 (520–807)	1254 (980–1528)	849 (720–978)	<0.001
CD8 count (cells/μL)	994 (675–1312)^a^	884 (645–1124)	567 (428–706)	458 (372–544)	0.001
Nadir CD4 (cells/μL)	180 (121–240)	266 (171–360)	−	−	0.392
HIV RNA (copies/mL)	26 (16–38)	27 (15–40)	−	−	0.757
Time since HIV diagnosis (months)	200 (153–248)	172 (112–232)	−	−	0.740
Treatment duration (months)	143 (116–170)	124 (89–159)	−	−	0.394
Fasting BG (mmol/L)	8.0 (6.9-9.0)^b,c^	5.3 (5.0-5.4)	9.0 (7.9-10.3)	5.3 (5.1-5.6)	<0.001
HbA1c (mmol/mol)	48 (44–52)^b,d^	35 (33–37)	60 (54–65)	36 (32–39)	<0.001
BMI	26 (24–28)^a,d^	25 (23–27)	28 (27–30)	25 (24–26)	0.007
Smoking (%)	44	24	23	21	0.233
Systolic BP (mmHg)	132 (125–139)	127 (121–133)	138 (131–144)	135 (130–140)	0.115
Diastolic BP (mmHg)	82 (77–87)	80 (77–84)	86 (82–91)	82 (79–85)	0.317
CVD10 (Framingham Risk Score)	29 (22–35)^c^	14 (10–18)	25 (19–30)	16 (12–20)^a^	<0.001

Infection with hepatitis C virus (HCV) was not an exclusion criterion in this study. However, only two participants were infected with HCV one in the HIV + T2D+ and one in HIV + T2D- group.

### Increased inflammation in HIV + T2D+ compared to HIV + T2D-

**Fig. 1:**
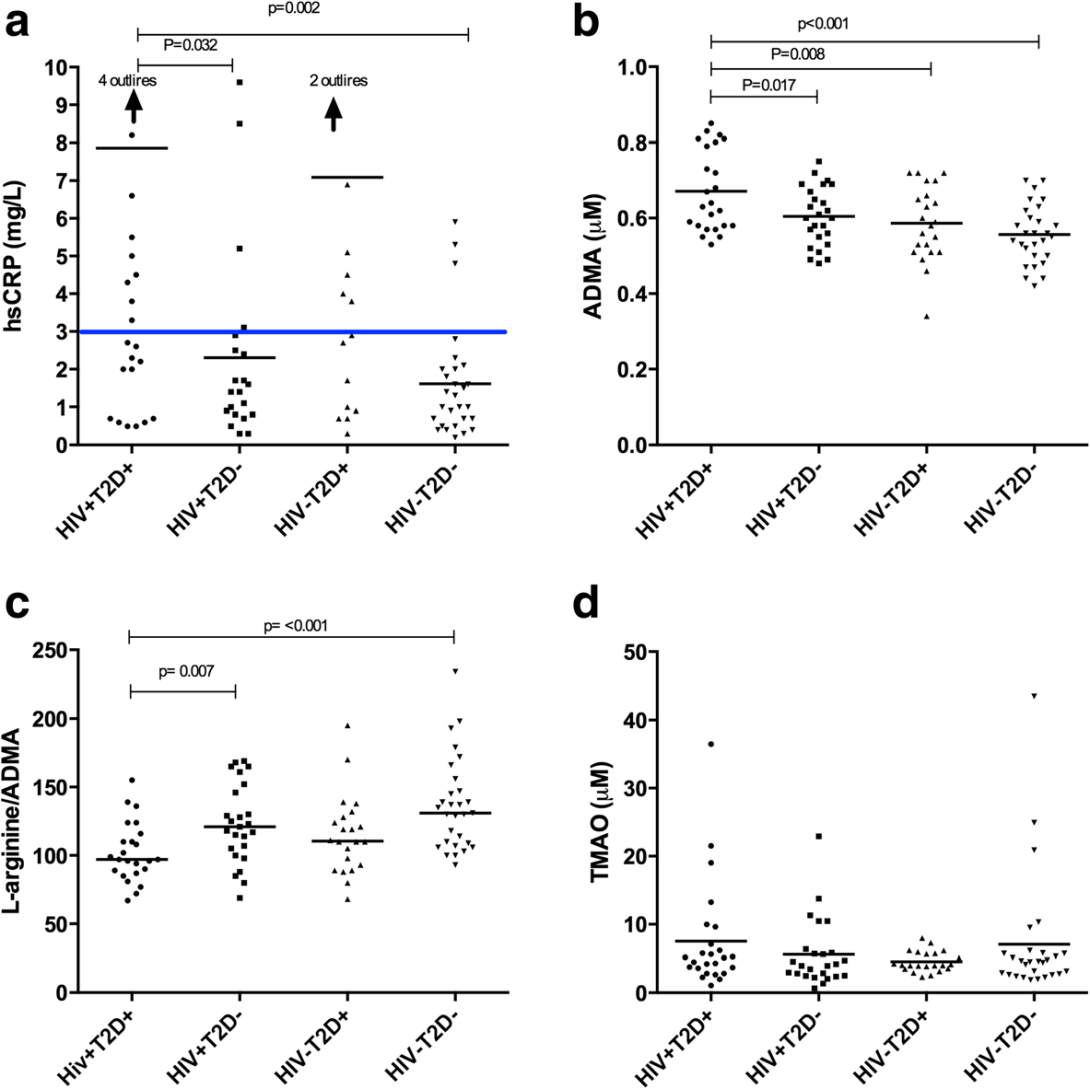
High sensitive C-reactive protein (hsCRP), Asymmetric dimethylarginine (ADMA), L-arginine/ADMA ratio and Trimethylamine N-oxide (TMAO) levels in the four groups: HIV-infected persons with type 2 diabetes (HIV + T2D+), HIV-infected persons without type 2 diabetes (HIV + T2D-), uninfected persons with type 2 diabetes (HIV-T2D+) and healthy controls(HIV-T2D-). Differences between groups were analyzed using one-way anova followed by t test. **a** hsCRP. Blue line shows a cut-off at 3 mg/L indicating high risk of CVD. For statistical analysis logarithmic transformation was used on data on hsCRP. The figure shows data before logarithmic transformation. **b**: ADMA, **c** L-arginine/ADMA ratio and **d** TMAO

HIV + T2D+ had a higher hsCRP compared to HIV + T2D- and HIV-T2D- (3.2 (0.86-12.0) vs. 1.5 (0.6-3.8) *p* = 0.032 and 1.1 (0.48-2.8) *p* = 0.002, respectively) but not compared to HIV-T2D+ (3.2 (0.86-12.0)) vs. 2.8 (0.48-2.8), *p* = 0.860). The percentage of HIV + T2D+, HIV + T2D-, HIV-T2D+ and HIV-T2D- with hsCRP above cut-off (3 mg/L) was 50%, 19%, 47% and 11%, respectively (Fig. 1a). More participants with HIV + T2D+ had hsCRP above cut-off compared to HIV + T2D- and HIV-T2D- but not compared to HIV-T2D+ (*p* = 0.05, *p* = 0.003 and *p* = 0.935, respectively).

To further investigate potential predictors of inflammation, a multivariate linear regression analysis was performed adjusting for age, gender, group (HIV + T2D+, HIV + T2D-, HIV-T2D+, and HIV-T2D-), BMI, CD4 count, and current smoking. The analysis was performed on the total study population (*n* = 100). Both group variable and current smoking were associated with hsCRP in multivariate analyses (*p* = 0.031 and *p* = 0.010, respectively) (Table 2). Adjusting for TMAO did not alter this association. Correlations between hsCRP and TMAO were not found in the total population or in any of the groups (data not shown).

**Table 2:** BMI:Body Mass Index, TMAO: Trimethylamine-N-oxide

Multiple Linear Regression Models with ADMA and hsCRP as Dependent Variables						
Characteristics	Unstandardized Coefficient β (95% CI) Model with ADMA	t	P	Unstandardized Coefficient β (95% CI) Model with hsCRP	t	P
Group	0.033 (0.016 to 0.049)	3.990	<0.001	0.104 (0.010 to 0.198)	2.202	0.031
Age	0.001 (−0.002 to 0.004)	0.771	0.443	0.010 (−0.007 to 0.026)	1.164	0.248
Gender	−0.048 (−0.104 to 0.007)	−1.732	0.087	−0.009 (−0.366 to 0.348)	−0.051	0.960
BMI	0.002 (−0.003 to 0.006)	0.746	0.457	0.007 (−0.022 to 0.036)	0.471	0.639
Smoking	−0.005 (−0.046 to 0.036)	−0.234	0.816	0.323 (0.081 to 0.566)	2.651	0.010
CD4+ cell count	-4.256E-5 (0.000 to 0.000)	−1.607	0.112	8.914 E-5 (0.000 to 0.000)	0.576	0.567
LogTMAO	0.088 (0.029 to 0.147)	2.953	0.004	−0.120 (−0.462 to 0.222)	−0.698	0.487

### Higher ADMA in HIV + T2D+ compared to HIV + T2D-

HIV + T2D+ had higher concentration of ADMA compared to HIV + T2D-, HIV-T2D+, and HIV-T2D- (0.67 μM (0.63-0.72) vs. 0.60 μM (0.57-0.64), *p* = 0.017 and 0.57 μM (0.51-0.63), *p* = 0.008 and 0.55 μM (0.52-0.58), *p* < 0.001, respectively) (Fig. 1b).

L-arginine/ADMA ratio was lower in HIV + T2D+ compared to HIV + T2D- and HIV-T2D- (102 (93–111) vs. 123 (111–135), *p* = 0.007) and 138 (125–151), *p* < 0.001, respectively), but not compared to HIV-T2D+ (102 (93–111) vs. (115 (102–128), *p* = 0.109 (Fig. 1c).

To further investigate potential predictors of endothelial dysfunction, a multivariate linear regression analysis was performed adjusting for age, gender, group (HIV + T2D+, HIV + T2D-, HIV-T2D+, and HIV-T2D-), BMI, CD4 count, and current smoking. The analysis was performed on the total study population (*n* = 100). The group variable predicted endothelial dysfunction (*p* < 0.001) (Table 2). In addition, we found a significant interaction between TMAO and group on elevated ADMA level, with TMAO being closely associated with ADMA in HIV + T2D+ (Additional file 1: Table S3). When adding TMAO to the multivariate model, TMAO was also independently associated with ADMA (Table 2). Of note, the beta coefficient of group status in predicting ADMA levels remained unchanged.

### Correlation between ADMA and TMAO in HIV infection

No differences in concentration of TMAO between groups were found (Fig. 1d). However, in the total population, there was a moderate positive correlation between TMAO and ADMA (r_s_ = 0.32, *p* = 0.001), which was driven by a strong correlation in HIV + T2D+ (r_s_ = 0.63, *p* = 0.001), and not significant in the other groups (HIV + T2D-: r_s_ = 0.35, *p* = 0.088, HIV-T2D+; r_s_ = −0.20, *p* = 0.374, HIV-T2D-; r_s_ = 0.20, *p* = 0.305).

## Discussion

Both HIV infection and T2D are characterized by chronic inflammation and increased risk of CVD. Little is known about the combined effect of HIV and T2D. In this study, higher ADMA and lower L-arginine/ADMA ratio was found in HIV + T2D+ compared to all three control groups indicating endothelial dysfunction. Furthermore, we found that 50% of HIV + T2D+ had hsCRP above 3 mg/L indicating an increased risk of CVD [26]. In contrast, only 19% of HIV + T2D- had hsCRP above cut-off. Interestingly, HIV + T2D+ displayed comparable level of inflammation compared to HIV-T2D+. Concentration of TMAO was comparable in the four groups, but a positive correlation between ADMA and TMAO was found mainly to be driven by persons with HIV + T2D+ suggesting an association between gut microbiota and endothelial dysfunction in this group.

Higher ADMA and lower L-arginine/ADMA ratio were found in patients with both HIV infection and T2D, indicating increased endothelial dysfunction in patients with both HIV infection and T2D. Importantly, only disease group was associated with ADMA in a multivariate linear regression model minimizing the risk of possible confounders. This finding may be of clinical relevance since studies in HIV-infected populations have found elevated ADMA to be independently associated with pulmonary arterial hypertension and increased coronary artery calcium score [28, 29]. Furthermore, in the setting of T2D, elevated ADMA was found to be associated with macrovascular disease [30]. Finally, ADMA was previously shown to enhance the predictive value of hsCRP for CVD in patients with T2D [31].

A recent study found elevated ratio between ADMA and systemic dimethylarginine (SDMA) in HIV infection. SDMA is produced from the same substrate as ADMA but not metabolized by the same enzyme [11, 32]. This finding indicates that the mechanism of ADMA accumulation in HIV infection is related to reduced activity of the enzyme dimethylarginine dimethylaminohydrolase which is responsible for metabolism of ADMA. This may be a consequence of oxidative stress generated from chronic inflammation. This is in agreement with our findings of elevated ADMA and hsCRP in patients with HIV + T2D+.

Chronic inflammation is associated with increased risk of CVD in the general population and in HIV infection [33–35]. In a multivariate linear regression model both smoking and disease group were associated with hsCRP. It is well known that smoking is associated with inflammation in both HIV-infected and uninfected persons [36, 37]. In the “Simple Trial Comparing Two Strategies for Management of Anti-Retroviral Therapy” (SMART study), elevated inflammation was found in HIV-infected persons when compared to the general population [38]. In our study, difference in hsCRP between HIV + T2D- and HIV-T2D- was not found, possibly due to low sample size or to a well-treated population receiving ART. In contrast, inflammation seemed closely linked to T2D. Thus, a very high percentage of participants with T2D (both with and without HIV infection) had hsCRP above cut-off indicating both that chronic inflammation is an essential part in the pathogenesis of T2D in persons with and without HIV-infection and a high risk of CVD in both groups. An independent association between chronic inflammation and increased risk of developing T2D is well established [39]. Furthermore, obesity is a well-known risk factor for developing T2D, and studies have shown that obesity and chronic inflammation are tightly linked [40]. This fits well with our finding of elevated BMI in HIV-T2D+. In contrast, no difference in BMI was found in HIV + T2D+ compared to HIV + T2D- and HIV-T2D-, indicating different pathogenesis leading to T2D in HIV-infected and uninfected persons. We speculate that HIV infection and the resulting chronic inflammation alter the balance between insulin sensitivity and insulin production. However, this is speculative and cannot be validated in a cross-sectional study.

Formation of TMAO depends on both dietary intake containing choline or carnitine and on the composition of the gut microbiota [19]. Elevated TMAO has been reported to be associated with cardiovascular events in persons with T2D as well as in the general population [19, 20, 22]. In a recent study of HIV-infected individuals, TMAO was increased in patients with myocardial perfusion defects. However no association to first-time myocardial infarction was found [41]. In our population, concentration of TMAO was comparable in all groups. This is consistent with recent studies reporting no difference in TMAO between HIV infected persons and healthy controls or persons with T2D and healthy controls [41, 42]. However, a larger study reported higher TMAO in persons with diabetes compared to healthy controls [20], and consensus has not been reached. In the present study, a positive correlation between TMAO and ADMA was observed. TMAO interferes with reverse cholesterol transportation and promotes foam cell formation and atherosclerosis [43]. Since endothelial dysfunction is an early step in the atherosclerotic process, this association is not surprising and it fits well with our previous finding of a close association between soluble CD14 and ADMA in HIV-infected persons on cART [23]. However, it is intriguing that the correlation was driven by the group of HIV + T2D+, and the association was not significant in either HIV + T2D- or HIV-T2D+. It may indicate an interacting effect of the two diseases possibly mediated by alterations in the gut microbiota. Further research to determine the possible clinical impact of this is warranted.

Inflammation is a possible link between TMAO and CVD. However, no correlations were found between hsCRP and TMAO. To our knowledge no other studies have investigated hsCRP and TMAO in HIV infection or T2D. Our finding is consistent with recent studies in healthy individuals [44, 45]. Albeit, an association between TMAO and TNF-α has been found [45]. Hence, the relation between TMAO and inflammation remains unclear.

The present study is limited by sample size and the cross sectional design, and it is not possible to conclude on causal relationship. Furthermore, the study is limited by the lack of HIV testing in the uninfected control groups. All participants in the uninfected control groups were without any symptoms, and the prevalence of HIV infection in Denmark is low (0.1%). Thus, it seems reasonable to assume these participants were uninfected. However, it would have been more accurate if an HIV test had been performed on all participants when included in the study. Unfortunately, plasma was only available for hsCRP measurements in 87 participants. The proportion of participants with hsCRP measurements was comparable in the four groups. However, the missing data represents a limitation to the study. The prevalence of smoking in the group of HIV + T2D+ seemed to be higher than in the other groups, although this was not significant. Since smoking was associated with inflammation this may be a limitation regarding our data on hsCRP. Also, ADMA is a relatively novel biomarker and little data on clinical endpoints exists. However, a large study in uninfected participants from The Framingham Offspring Cohort found that ADMA and L-arginine/ADMA ratio was associated with all-cause mortality after a follow-up period of 10.9 years [46]. A recent systemic review, including 22 prospective studies, found ADMA associated with CVD [47]. Nevertheless, comparisons between the well-defined and highly prevalent disease categories included in this study are, to our knowledge, novel. Larger prospective studies to determine if ADMA is associated with clinical endpoints in HIV-infected persons, especially in HIV-infected persons with T2D, would be very interesting.

## Conclusions

In conclusion, our findings imply that elevated inflammation and endothelial dysfunction could be factors contributing to elevated risk of CVD in HIV-infected persons with T2D, compared to HIV- infected persons without T2D. The possible combined effect of HIV infection and T2D, and the potential role of gut microbiota in this regard, should be further investigated. With an aging HIV-infected population, lifestyle diseases and co-morbidity are emerging clinical challenges. This highlights the importance of CVD preventive strategies and close clinical monitoring for cardiovascular risk factors in HIV-infected persons with T2D. Finally, research targeting possible anti-inflammatory treatments to reduce chronic inflammation in HIV-infected persons is warranted.

## Additional file

Additional file 1: Table S3. HIV+T2D+: HIV infected persons with type 2 diabetes, TMAO: Trimethylamine-N-oxide. (PPTX 46 kb)

## Abbreviations

ADMA: Asymmetric dimethylarginine
BMI: Body mass index
cART: Combination anti-retroviral therapy
CVD: Cardiovascular disease
HIV: Human immunodeficiency virus
HsCRP: High sensitive C-reactive protein
SDMA: Systemic dimethylarginine
T2D: Type 2 diabetes
TMAO: Trimethylamine-N-oxide
