# Supplementary material for: HIV-infected persons with type 2 diabetes show evidence of endothelial dysfunction and increased inflammation
Source: BMC Infect Dis. 2017 Mar 29;17:234. doi: 10.1186/s12879-017-2334-8 (PMC5372333; doi:10.1186/s12879-017-2334-8)
Supplement: Additional file 1: Table S3. — HIV+T2D+: HIV infected persons with type 2 diabetes, TMAO: Trimethylamine-N-oxide. (PPTX 46 kb) [file 12879_2017_2334_MOESM1_ESM.pptx]

## Slide 1
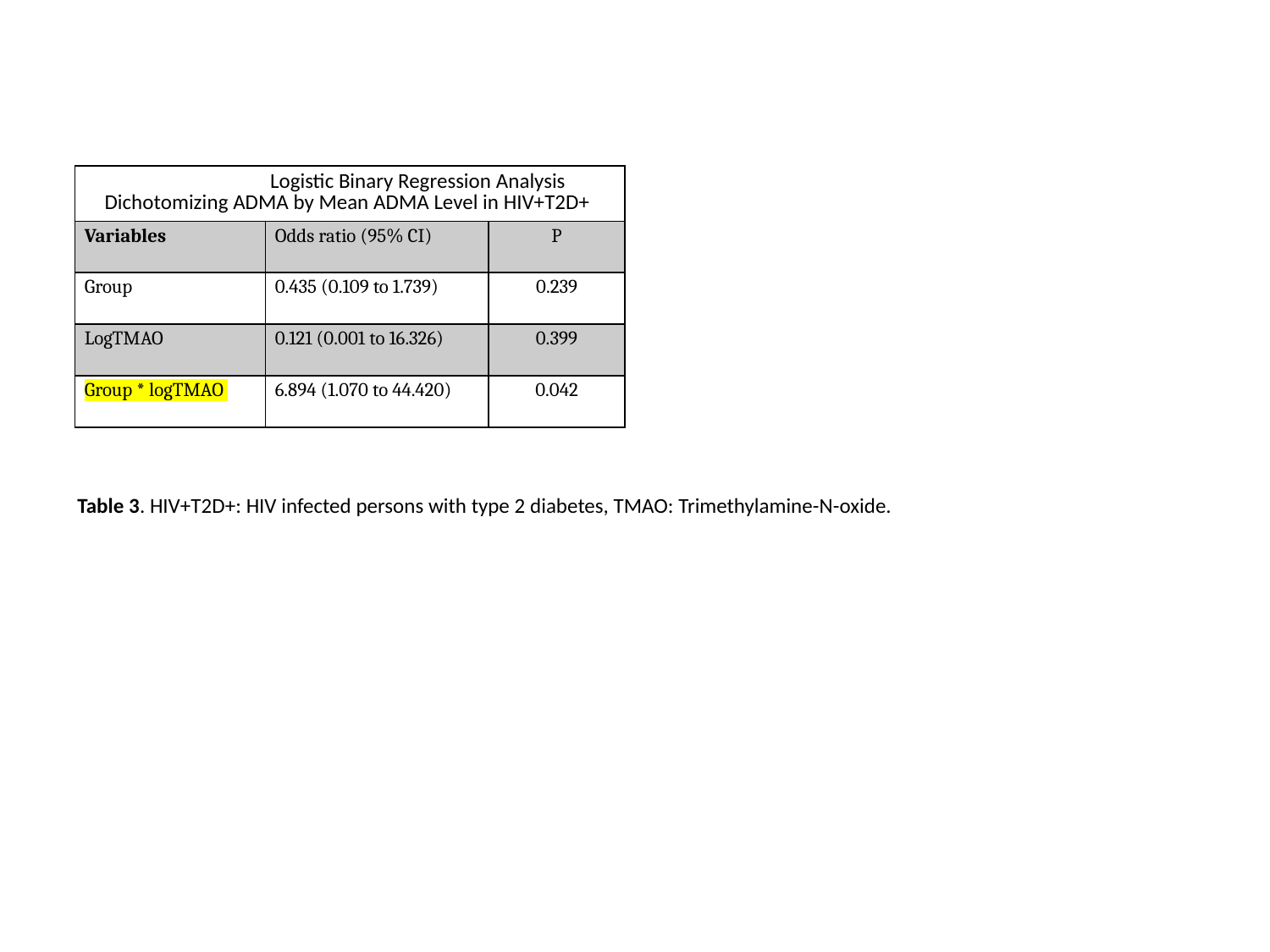

| Logistic Binary Regression Analysis Dichotomizing ADMA by Mean ADMA Level in HIV+T2D+ | | |
| --- | --- | --- |
| Variables | Odds ratio (95% CI) | P |
| Group | 0.435 (0.109 to 1.739) | 0.239 |
| LogTMAO | 0.121 (0.001 to 16.326) | 0.399 |
| Group \* logTMAO | 6.894 (1.070 to 44.420) | 0.042 |
Table 3. HIV+T2D+: HIV infected persons with type 2 diabetes, TMAO: Trimethylamine-N-oxide.
